# Expression Profiling of Circulating Tumor Cells in Pancreatic Ductal Adenocarcinoma Patients: Biomarkers Predicting Overall Survival

**DOI:** 10.3389/fonc.2019.00874

**Published:** 2019-09-10

**Authors:** Consuelo Amantini, Maria Beatrice Morelli, Massimo Nabissi, Francesco Piva, Oliviero Marinelli, Federica Maggi, Francesca Bianchi, Alessandro Bittoni, Rossana Berardi, Riccardo Giampieri, Giorgio Santoni

**Affiliations:** ^1^School of Biosciences and Veterinary Medicine, University of Camerino, Camerino, Italy; ^2^School of Pharmacy, Experimental Medicine Section, University of Camerino, Camerino, Italy; ^3^Department of Specialistic Clinical and Odontostomatological Sciences, Polytechnic University of Marche, Ancona, Italy; ^4^Department of Molecular Medicine, Sapienza University of Rome, Rome, Italy; ^5^Oncology Clinic, AOU Ospedali Riuniti, Polytechnic University of Marche, Ancona, Italy

**Keywords:** circulating tumor cells, pancreatic cancer, overall survival, gene signature, digital droplet PCR, atypical CTC

## Abstract

The interest in liquid biopsy is growing because it could represent a non-invasive prognostic or predictive tool for clinical outcome in patients with pancreatic ductal adenocarcinoma (PDAC), an aggressive and lethal disease. In this pilot study, circulating tumor cells (CTCs), CD16 positive atypical CTCs, and CTC clusters were captured and characterized in the blood of patients with PDAC before and after palliative first line chemotherapy by ScreenCell device, immunohistochemistry, and confocal microscopy analysis. Gene profiles were performed by digital droplet PCR in isolated CTCs, five primary PDAC tissues, and three different batches of RNA from normal human pancreatic tissue. Welsh's *t*-test, Kaplan-Meier survival, and Univariate Cox regression analyses have been performed. Statistical analysis revealed that the presence of high CTC number in blood is a prognostic factor for poor overall survival and progression free survival in advanced PDAC patients, before and after first line chemotherapy. Furthermore, untreated PDAC patients with CTCs, characterized by high ALCAM, POU5F1B, and SMO mRNAs expression, have shorter progression free survival and overall survival compared with patients expressing the same biomarkers at low levels. Finally, high SHH mRNA levels are negatively associated to progression free survival, whereas high vimentin mRNA levels are correlated with the most favorable prognosis. By hierarchical clustering and correlation index analysis, two cluster gene signatures were identified in CTCs: the first, with high expression of VEGFA, NOTCH1, EPCAM, IHH, is the signature of PDAC patients before chemotherapy, whereas the second, with an enrichment in the expression of CD44, ALCAM, and POU5F1B stemness and pluripotency genes, is reported after palliative chemotherapy. Overall our data support the clinic value of the identification of CTC's specific biomarkers to improve the prognosis and the therapy in advanced PDAC patients.

## Introduction

Pancreatic ductal adenocarcinoma (PDAC) is an aggressive and lethal disease whose incidence rate is growing. The overall survival (OS) rate at 5 years is only 9%, the lowest percentage respect to other cancers (1). After diagnosis only 24% of patients survive 1 year and the 85% of them die within 5 years from diagnosis (2). This high death rate depends not only on the development of drug resistance, but also on late diagnosis. The majority of PDAC patients are treated with a first line palliative chemotherapy to reduce symptoms and prolong their survival (3). However, data obtained using combined therapy with gemcitabine plus paclitaxel or folfirinox, have demonstrated an increase in the chemotherapeutic efficacy (4, 5).

In PDAC, the development of metastases occurs very early and their presence is discovered already during the first diagnosis. Metastases represent the main cause of cancer-related deaths and the mechanisms of the metastatic spread are not yet well-known. Recently, the detection/isolation of circulating tumor cells (CTCs) in blood samples from cancer patients prompted high interest. CTCs, believed to be responsible for seeding and dissemination of cancer, originate from the primary tumor mass and spread in the peripheral circulation among immune cells and erythrocytes (6). In addition, CTCs are also able to aggregate forming clusters, termed circulating tumor microemboli, whose size and concentration have been found to influence the development of metastases. It is now accepted that CTC clusters have survival advantage in the circulation, since the aggregation protect tumor cells from apoptosis, shear stress, and immune response facilitating the colonization (7). Thus, CTCs have been utilized as prognostic or predictive tool for clinical outcome in patients with localized, metastatic and recurrent disease and the CTC number is now considered a prognostic factor in breast, colorectal, prostate, and lung cancers (8).

Among the different technologies employed to isolate and purify CTCs, the ScreenCell® microfiltration is an epitope-independent size-based device, able to capture CTCs, both EPCAM positive and negative. It has been used in CTC identification in rare tumors like hemangiopericytoma (9) as well as in more common tumors such as non-small cell lung, bladder, prostate, head, and neck cancers (10–13) including PDAC (14).

Several studies demonstrated that a large number of CTCs and circulating tumor microemboli is detected in blood samples from PDAC patients with high accuracy and this appears to be clinically relevant avoiding the need of invasive tumor biopsies (8, 15, 16). In this regard, Nagrath et al. by using the CTC-chip on blood samples from PDAC patients, identified the presence of CTCs in the 100% of cases and the number of CTCs detected ranged from 9 to 831/ml (17). CTCs are found in the blood of patients with all PDAC stages and their presence is associated with poor progression-free survival (PFS), shorter OS, liver metastases, and poor tumor differentiation (18–20). Remarkably, high number of CTCs and unfavorable number of CTC clusters are associated with a trend for short OS (21).

Moreover, the recurrence occurs earlier in patients with CTCs than those without them, suggesting that CTCs are involved in pancreatic cancer malignancy and can be used to predict outcome and prognosis (18, 20). In addition, preclinical studies, performed using a xenograft mouse model of pancreatic adenocarcinoma, demonstrated that CTC concentration is markedly reduced in the pharmacologically treated group compared to the untreated one, indicating CTCs as a promising biomarker to monitor treatment efficacy (22). Interestingly, a recent report showed that in the blood of PDAC patients, the CTC population is represented not only by cancer cells but also by atypical CTCs that are hybrid cells, also called tumacrophages, deriving from the fusion between macrophages and cancer cells (23, 24). It has also been shown that the presence of tumacrophages significantly correlates with advanced PDAC disease (23).

At present, little is known about the neoplastic features, clinical significance, and molecular profiles of CTCs in PDAC patients. Several altered signaling pathways have been found in pancreatic cancers as KRAS, EGFR, NOTCH, WNT, and Hedgehog signaling pathways (25, 26). However, specific biomarkers useful for the early detection or for predicting treatment response are still missing. The complexity to manage clinical situations absolutely needs of new therapeutic strategies and further efforts must be made to identify novel targets for the development of personalized treatment options (27). In addition, given the anatomical difficulty of reaching the primary site of the tumor, it is problematic to monitor disease progression by invasive repeated biopsies. Thus, a multimarkers' analysis represents a good strategy to better understand the features of CTCs in terms of aggressiveness and phenotype. This could make it possible to select the most effective treatment and to facilitate a personalized therapy.

The aim of this study was to evaluate the expression of different genes involved in several signaling pathways in CTCs, isolated from patients with metastatic PDAC, in order to correlate the gene expression profiles with clinical parameters, before and after the palliative chemotherapeutic treatments.

## Materials and Methods

### Patient Recruitment and Sample Processing

The study population consisted of patients with a histologically/cytologically confirmed diagnosis of metastatic/locally advanced PDAC, who were candidates to receive 1st line palliative chemotherapy (*n* = 20) and with ages between 44 and 76, hospitalized from 2016 to 2018 at Università Politecnica delle Marche–Azienda Ospedaliero-Universitaria Ospedali Riuniti Umberto I—Lancisi—Salesi, Ancona, Italy. Scheduled evaluations of disease status were performed via computed tomography scan of the chest and abdomen. Database with demographic, pathologic, and relevant clinical outcome/survival variables was maintained in a prospective manner. RECIST 1.1 criteria were used to evaluate the radiological responses to treatment, at approximately 3 months after the beginning of 1st line chemotherapy and every 3 months thereafter. Data regarding OS (time between the diagnosis and the death or lost-at-follow-up visit), OS1 (the time between the 1st cycle of chemotherapy and death or lost-at-follow-up visit) and PFS (the time between the 1st cycle of chemotherapy and the 1st radiological progression or lost-at-follow-up-visit) were collected. All patients gave their consent prior to blood draws and the local Ethical Committee approved the study.

Peripheral blood samples from patients (6 ml) were collected in a K2-EDTA tube, before and after 3 months the beginning of the chemotherapy. The blood samples were processed within 3 h by using ScreenCell devices (Sarcelles, France) according to the protocol with some modifications to better eliminate peripheral blood cells. Briefly, after filtration, ScreenCell filters were washed with RPMI 1640 medium and then with Red Blood Lysis Buffer (Milteny Biotec, Bologna, Italy). The isolated cells were then detached from the filter by pipetting, collected in RPMI medium and the resulting cell suspension was filtered again. Blood samples from 5 healthy donors were processed as negative control.

### Cell Counting

CTCs, collected in the second filter, were observed by stereo-microscope with bright-field illumination. Two independent operators performed a blind evaluation for each sample of the selected isolated cells, dividing patients in two categories: those with more than 10 CTCs and those with less. The presence of CTC clusters was also assessed and patients were divided in positive (Yes) or negative (No) for this parameter.

### RNA Extraction, Reverse Transcription, and Digital Droplet PCR (ddPCR)

Total RNA from isolated CTCs was extracted by using the Single Shot Cell Lysis Kit (Bio-Rad, Hercules, CA, USA) according to the protocol. As control, three different total RNAs from normal pancreas tissues were purchased (OriGene Technologies, Rockville, MA, USA) and total RNA was extracted from 5 different primary PDAC tissue specimens, not autologous to the patients from whom CTCs were isolated (from Università Politecnica delle Marche–Azienda Ospedaliero-Universitaria Ospedali Riuniti Umberto I—Lancisi—Salesi, Ancona), by “RNeasy® FFPE” kit (QIAGEN, Milan, Italy).

Total RNA was retro-transcribed by Iscript Advanced cDNA Synthesis kit (Bio-Rad) and the resulting cDNA was used to pre-amplify each sample for all primers used in the gene expression analysis by SSOADvancedPreAmp Kit and PrimePCRPreAMP Assays (Bio-Rad). The ddPCR Supermix for Probes (No dUTP) (Bio-Rad) and the specific PrimePCR™ ddPCR™ Expression Probe Assays conjugated with FAM or HEX fluorescent dyes (the same pool used in the pre-amplification step) (Bio-Rad) were then used to perform the ddPCR. The analyzed target genes were: CD44, DHH, ALCAM, IHH, VEGFA, NOTCH1, VEGFB, PTCH1, ZEB1, PTCH2, ZEB2, SHH, EPCAM, SMO, POU5F1B, SPARC, STAT3, vimentin (VIM), and NOTCH2. Data, normalized to β-actin concentration, were analyzed using the QuantaSoft Software (Bio-Rad). Since some of the analyzed transcripts could also be expressed, although at low levels, in normal blood cells, ddPCR analysis was carried out identifying the gene expression values obtained from white blood cells and taking them as negative threshold.

After ddPCR, according to the ROC analysis performed before and after palliative 1st line chemotherapy, patients were sub-grouped for each gene in high (H) and low (L) expression.

Heat-maps were generated with hierarchical clustering analysis by the software Multi Experiment Viewer (MeV) Version 4.9.0. To compare CTCs with PDAC biopsies, gene expression levels were expressed as fold changes respect to normal pancreas RNAs used as calibrator.

### Immunohistochemistry

Purified CTCs were fixed by using paraformaldehyde (4%) for 5 min at room temperature. After washing with PBS, cells were permeabilized by using 0.3% Triton X-100 in PBS for 15 min at room temperature. To block endogenous peroxidase, samples were incubated with 0.3% H_2_O_2_ for 15 min and then the blocking solution (3% BSA, 0.3% Triton X-100 in PBS) was used for 60 min at room temperature. Thereafter, cells were firstly incubated with anti-human pancytokeratin antibody (pan-CK, 1:50, DAKO, Agilent, Santa Clara, CA, USA) overnight at 4°C and then with anti-mouse biotin-conjugated secondary antibody for 30 min (1:200, ThermoFisher Scientific, Waltham, MA, USA). The immunodetection was performed using the VECTASTAIN® Elite® ABC System (Vectastain Laboratories, Burlingame, CA, USA), according to the provided protocol and counterstaining with hematoxylin for 30 s. Four random fields of each filter were analyzed under 40X magnification using the Olympus BX51 Microscope and the ImageJ software (National Institutes of Health, Bethesda, MD, USA).

### Confocal Microscope Analysis

Isolated CTCs were fixed with 4% paraformaldehyde for 5 min at room temperature and permeabilized as above described. For epithelial markers analysis, cells were stained with mouse anti-human pan-CK (1:50, Agilent), anti-human EPCAM (1:50, Cell Signaling Technology, Danvers, MA, USA), anti-human CD45, and anti-human CD16 (Cell Signaling Technology) antibodies followed by goat anti-mouse secondary antibody Alexa 594 (1:100, ThermoFisher Scientific), labeled with DAPI (ThermoFisher Scientific) and examined under 40X magnification using the Confocal Microscopy Nikon C2plus and the NIS software (Nikon, Otawara, Japan).

### Statistical Analysis

This study was an exploratory research. Statistical analysis was performed by using the Welch's *t*-test (GraphPad). Patients were divided in two groups according to: high and low CTC/cluster number, high and low gene expression levels. In addition, the Welch's *t*-test was used to compare gene expression levels between PDAC biopsy and CTCs. *p* < 0.05 was considered as statistically significant. The Kaplan-Meier (KM) method was also used for survival analysis. For Univariate analysis of significance (MedCalc package, MedCalc® v16.4.3), the long-rank test or Cox analysis was used. *p* < 0.05 was considered as statistically significant.

We determined, by Relative Operating Characteristic (ROC) curve analysis, the expression value for each analyzed gene (cDNA copies/μl) that best discriminates between good and poor prognosis.

For hierarchical clustering, we applied the most common settings that is “average linkage” as agglomeration rule and Pearson correlation to measure the similarity among gene profiles.

The analysis of frequency distribution was performed using Chi-squared test selecting as expected frequencies <60 for age, male category for sex, yes for lymph node invasion, yes for distant metastasis and high for CTC number; *p* < 0.05 was considered as statistically significant. To study the survival time we considered: OS, OS1, and PFS.

## Results

### Capture and Detection of CTCs From Blood of PDAC Patients

All the 20 patients, enrolled in this study, had histologically confirmed diagnosis of PDAC; the list of patients' characteristics, including average age, sex, TNM classification, chirurgical resection, and 1st line chemotherapy options, is shown in Table 1. The median OS, OS1, or PFS of the patient population were 11.87, 8.75, and 6.16 months, respectively. The KM analysis was carried out to evaluate OS and PFS in relation to the clinic-pathological characteristics of PDAC patients. No statistical significance was found among age, TNM stage, different 1st line palliative chemotherapy protocol and OS, OS1, and PFS. Sex showed positive correlation with PFS (*p* = 0.0470) (Supplementary Table 1).

**Table 1:** Patient demographics and clinical features.

**Number of patients**	**20**
Average age	64.04 ± 8.07
Median age	64.13
Sex	F (4)
	M (16)
T	T1 (1)
	T2 (6)
	T3 (8)
	T4 (5)
N	Yes (16)
	No (4)
M	Yes (13)
	Liver (8)
	Lung (1)
	Peritoneum (1)
	Liver + lung (1)
	Liver + peritoneum (1)
	Lung + peritoneum (1)
	No (7)
Chirurgical resection	Yes (4)
	No (16)
First-line chemotherapy	Yes (19)
	Gemcitabine (4)
	Folfirinox (4)
	Gemcitabine + abraxane (11)
	No (1)

CTCs were isolated from blood samples in patients before chemotherapy (20 patients) and after standard palliative 1st line chemotherapy (19 samples, since one patient died during treatment).

**Figure 1:**
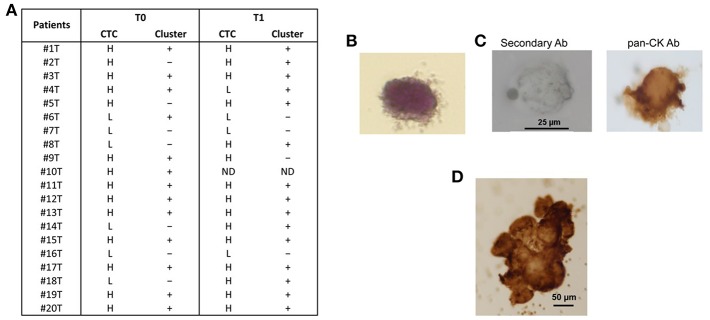
CTC isolation from blood samples of PDAC patients. **(A)** After isolation of CTCs on the ScreenCell filter, PDAC patients were classified, according to the evaluation performed by stereo-microscope, in H (High, more than 10 CTCs); L (Low, <10 CTCs);—(no presence of clusters); + (presence of clusters); ND (not determined). T0 = before chemotherapy; T1 = after chemotherapy. **(B)** Representative image of isolated CTC stained with H&E. **(C)** Representative image of single CTC in ScreenCell filter stained with anti-human pan-CK and processed by immunohistochemistry. The omission of the primary antibody was used as negative control. **(D)** Representative image of CTC cluster in ScreenCell filter processed as described in **(C)**.

Both single CTCs and/or CTC clusters were captured by microfiltration. In particular, before chemotherapy, low CTCs number (<10 CTCs/ml blood) was evidenced in 6/20 (30%) and high number (more than 10 CTCs/ml blood) in 14/20 (70%) of the PDAC patients; 3 months later, after palliative chemotherapy, low CTCs number was found in 4/19 (21%) and high in 15/19 (79%) PDAC patients. Moreover, since CTC clusters, composed by more than 3 cells, have a greater predisposition of forming distal metastasis than single CTCs (7), our attention was focused on the presence of CTC aggregates. CTC clusters were present in 13/20 (65%) patients before the chemotherapy and in 15/19 (79%) patients after palliative chemotherapy (Figure 1A). Neither single CTCs nor CTC clusters were found in the blood of healthy donors. After chemotherapy (T1), in three patients the number of CTCs was increased and in one was reduced. Regarding to CTC clusters, in five patients the number is increased and in other two they were reduced.

**Figure 2:**
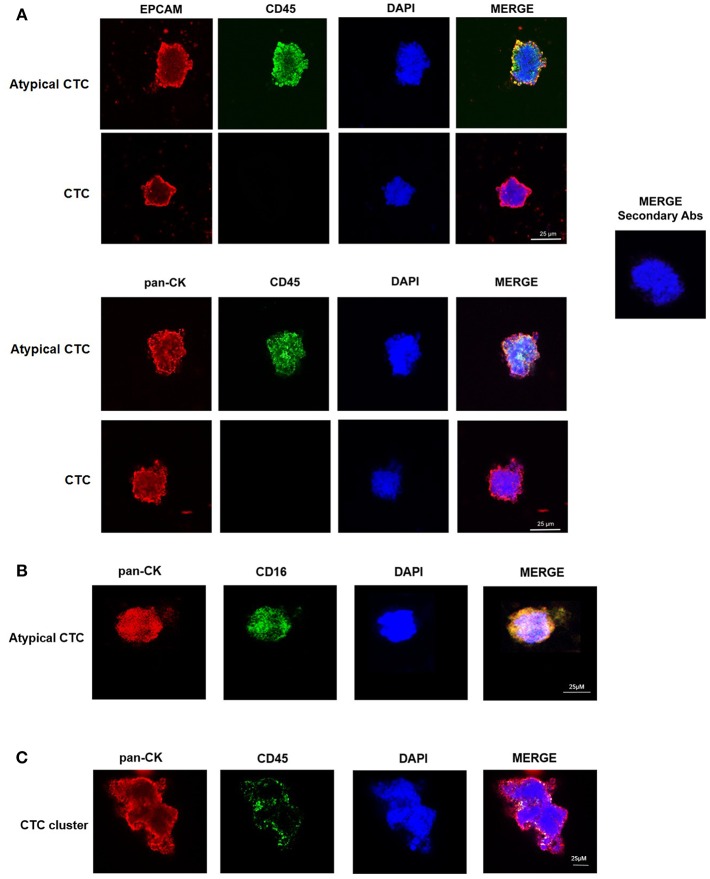
Confocal microscopy analyses of isolated CTCs. **(A)** Representative images of single CTC and atypical CTC stained with anti-human EPCAM or anti-human pan-CK and anti-human CD45 Abs. DAPI was used to counteract nuclei. The omission of the primary antibody was used as negative control. **(B)** Representative image of atypical CTC stained with anti-human pan-CK and anti-human CD16 Abs. DAPI was used to counteract nuclei. **(C)** Representative image of a CTC cluster stained with anti-human pan-CK and anti-human CD45 Abs. DAPI was used to counteract nuclei.

We confirmed the CTC phenotype by Hematoxylin and Eosin (H&E) staining, immunohistochemistry and confocal microscopy using anti-human pan-CK, anti-EPCAM, and anti-CD45 antibodies. The H&E staining evidenced, as previously described (15), that the single CTC displays a big size with a hyperchromatic nucleus larger than 14 μm and scant well-defined small rim of cytoplasm (Figure 1B). By immunohistochemistry, we showed that both single CTCs and CTC clusters, forming irregular microemboli, were markedly pan-CK^+^ (Figures 1C,D). Finally, the confocal microscopy analysis demonstrated the presence in the CTC population of both EPCAM^+^ or pan-CK^+^ CD45^−^ cancer cells and EPCAM^+^ or pan-CK^+^ CD45^+^ atypical CTCs (Figure 2A). Since it has been suggested that atypical CTCs derived from the fusion between cancer cells and macrophages (23), we also evaluated, in CTCs, the expression of CD16 found to be expressed by pro-tumorigenic macrophages (28). The isolated atypical CTCs expressed CD16 (Figure 2B) supporting the previous data about the origin of these hybrid cells. Finally, we demonstrated that CTC cluster is formed by both pan-CK^+^ CD45^−^ cancer cells and pan-CK^+^ CD45^+^ atypical CTCs (Figure 2C).

### Increased CTC Number Correlates With Poor Prognosis in PDAC Patients

**Figure 3:**
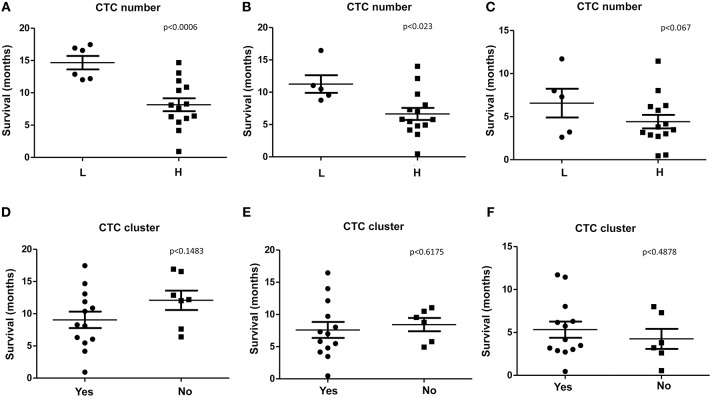
OS, OS1, and PFS according to CTC or CTC cluster number. **(A–C)** OS, OS1, and PFS were evaluated stratifying patients according to H (High, more than 10 CTCs/ml) or L (Low, <10 CTCs/ml) CTCs amount. **(D–F)** OS, OS1, and PFS, were evaluated stratifying patients according to presence (Yes) or not (No) of CTC clusters. *p* < 0.05 was considered as statistically significant.

We found that, PDAC patients with high number of CTCs/ml (H), evaluated before the chemotherapy (T0), display a significant shorter survival respect to patients with low number (L) when considering OS and OS1 (Figures 3A,B). Concerning PFS, a tendency toward significance was found between high and low CTCs number (Figure 3C). Our results suggest that high number of CTCs represents a negative factor for survival in PDAC patients. No significant correlation between the presence of CTC cluster (T0) and OS, OS1, or PFS was observed (Figures 3D–F).

### Gene Expression Profile of CTCs in PDAC Patients

The gene expression profile of CTCs from PDAC patients, before and after palliative chemotherapy, in PDAC biopsies and in normal pancreas RNAs, was evaluated.

**Figure 4:**
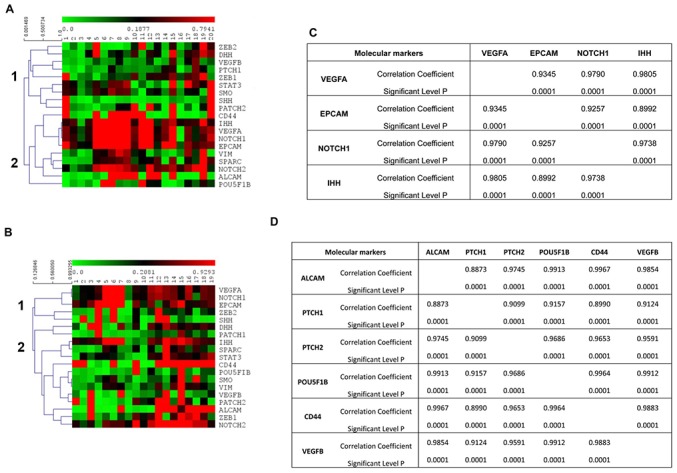
Gene expression profiles of CTCs from PDAC patients. **(A,B)** Hierarchical clustering was used to analyze the expression levels of 19 genes, assessed by ddPCR, in CTCs from PDAC patients before chemotherapy **(A)** and after palliative chemotherapy **(B)**. Two different main gene clusters were found, as shown with vertical bars and identified as 1 and 2, before chemotherapy (*p* < 0.0001) and after chemotherapy (*p* < 0.038). **(C,D)** Correlation matrix showing only the gene pairs whose expression levels were found to be positively correlated with a correlation coefficient more than 0.8500.

At first, we found that the 19 genes analyzed by ddPCR analysis were differently expressed and clustered in the same patients, before and after therapy, as shown by the heat-map (Figures 4A,B). In fact, according to the expression levels, two different main gene clusters were identified in the hierarchical clustering before therapy: ZEB2, DHH, VEGFB, PTCH1, ZEB1, STAT3, SMO, SHH, PTCH2 (cluster 1) vs. CD44, IHH, VEGFA, NOTCH1, EPCAM, VIM, SPARC, NOTCH2, ALCAM, POU5F1B (cluster 2) (Figure 4A). Whereas, after 3 months of chemotherapy, the gene map showed a different grouping: ZEB2, SHH, VEGFA, NOTCH1, EPCAM (cluster 1) vs. DHH, VEGFB, PTCH1, ZEB1, STAT3, SMO, CD44, IHH, VIM, SPARC, NOTCH2, ALCAM, POU5F1B, PATCH2 (cluster 2) (Figure 4B). Since the gene grouping, based on the expression profiles, changes after chemotherapy, our data suggest that the treatment is able to modify the gene expression levels in CTCs. The analysis of the correlation index for all studied genes (data not shown) also confirmed the presence of a strong association in the expression levels of specific genes and two different gene signature models for CTCs from PDAC patients were built before and after palliative chemotherapy, respectively. The VEGFA/EPCAM/NOTCH1/IHH network of functional genes marks the PDAC patients before chemotherapy (Figure 4C), whereas the VEGFB/ALCAM/PTCH1/2/POU5F1B/CD44 cluster characterizes CTCs from patients after the conditioned chemotherapy (Figure 4D).

**Figure 5:**
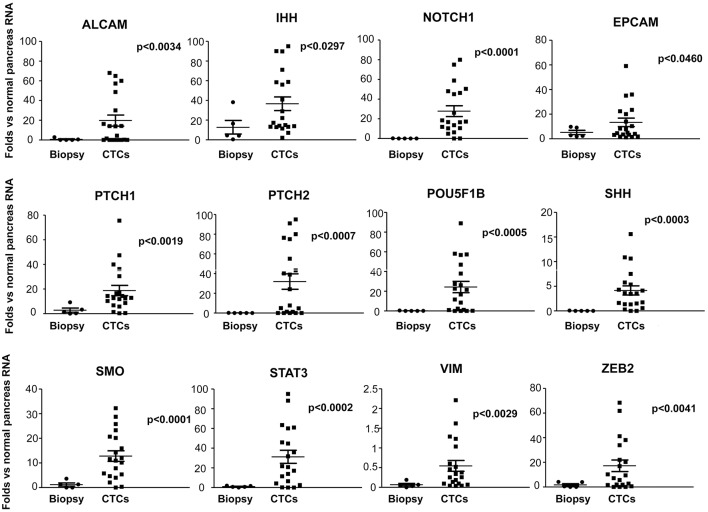
Up-regulated genes in PDAC CTCs respect to normal pancreas and PDAC biopsies. Genes found to be expressed at significant higher levels in CTCs respect to PDAC biopsies or normal pancreas RNA, are shown. Gene expression levels, evaluated by ddPCR, are expressed as fold changes respect to normal pancreas RNAs, used as calibrator. *p* < 0.05 was considered as statistically significant.

Moreover, since CTCs are implicated in the metastatic spread, to better understand the differences between circulating and primary tumor mass cells, we compared the gene expression levels, evaluated as fold changes respect to normal pancreatic RNAs, of CTCs (T0) with PDAC biopsies. A significantly increased expression of ALCAM, SHH, IHH, PTCH1, PTCH2, ZEB2, SMO, VIM, EPCAM, POU5F1B, STAT3, and NOTCH1 was found in CTCs respect to normal pancreatic RNA and, even more interestingly, compared with PDAC biopsies (Figure 5). Overall, these results suggest that the ability of CTCs to circulate is associated with the enhancement in the expression levels of several genes mainly involved in the Hedgehog, angiogenesis, epithelial mesenchymal transition (EMT) and transcription regulation pathways.

### Detection of Different EMT Phenotypes in CTCs From PDAC Patients

Among all the analyzed genes, our attention was focused on those strongly associated with the stemness and aggressiveness as well as EMT phenotype. On the basis of EMT markers (e.g., EPCAM/VIM) we demonstrated that about 40% of patients display epithelial CTCs (E-CTCs), whereas 60% show hybrid CTCs (H-CTCs) expressing both EPCAM and VIM (Table 2). No change in the percentage of patients showing E-CTCs was found 3 months after chemotherapy (42%), whereas a reduction of those with H-CTCs (37%) as well as the occurrence of patients (21%) with mesenchymal CTCs (M-CTCs) was observed (Table 3). We then analyzed the distribution of patients, according to the clinic-pathological features and the different EMT-phenotypes of CTCs, before and after palliative chemotherapy (Tables 2, 3). We found that before chemotherapy, the distribution of patients for age, gender, lymph node metastasis, distant metastasis, and CTC number was significantly different between E-CTC and H-CTC groups (Table 2); similarly, after palliative chemotherapy, as respect to lymph node metastasis and distant metastasis, E-, H-, and M-CTC groups were significantly different (Table 3). Moreover, 1/20 (5%) and 3/19 (16%) PDAC patients, before and after palliative chemotherapy, respectively, evidenced a more aggressive CTC phenotype, characterized by high levels of CD44/ALCAM (29).

**Table 2:** PDAC patient distributions according to EMT phenotypes of CTCs and clinic-pathological features before chemotherapy.

		**Epithelial CTC (%)**	**Hybrid CTC (%)**	
Age (year)	<60	5/20 (25%)	2/20 (10%)	
	>60	5/20 (25%)	8/20 (40%)	^*^
Gender	Male	10/20 (50 %)	8/20 (40%)	
	Female		2/20 (10%)	^*^
TNM stage	I	–	1/20 (5%)	
	II	2/20 (10%)	4/20 (20%)	
	III	5/20 (25%)	3/20 (15%)	
	IV	3/20 (15%)	2/20 (10%)	
Lymph node metastasis	Negative	1/20 (5%)	3/20 (15%)	
	Positive	9/20 (45%)	7/20 (35%)	^*^
Distant metastasis	Negative	5/20 (20%)	2/20 (10%)	
	Positive	5/20 (25%)	8/20 (40%)	^*^
Metastatis sites	Liver	4/13 (30.8%)	3/13 (23.1%)	
	Others	1/13 (7.6%)	5/13 (38.5%)	
CTC number	High	8/20 (40%)	7/20 (35%)	
	Low	1/20 (5%)	4/20 (20%)	^*^
CTC cluster	Yes	7/20 (35%)	6/20 (30%)	
	No	3/20 (15%)	4/20 (20%)	

* *p < 0.01*.

**Table 3:** PDAC patient distributions according to the different EMT phenotypes of CTCs and clinic-pathological features after palliative chemotherapy.

		**Epithelial CTC 8/19 (42.1%)**	**Hybrid CTC 7/19 (36.8%)**	**Mesenchymal CTC 4/19 (21.1%)**	
TNM stage	I		1/19 (5.3%)		
	II	3/19 (15.8%)	2/19 (10.1%)	1/19 (5.3%)	
	III	4/19 (21.1%)	3/19 (15.8%)		
	IV	1/19 (5.3%)	1/19 (5.3%)	3/19 (15.8%)	
Lymph node metastasis	Negative	3/19 (15.8%)	1/19 (5.3%)		
	Positive	6/19 (31.6%)	7/19 (36.9%)	2/19 (10.1%)	^*^
Distant metastasis	Negative	4/19 (21.1%)	1/19 (5.3%)	2/19 (10.1%)	
	Positive	4/19 (21.1%)	6/19 (31.6%)	2/19 (10.1%)	^*^
Metastasis sites	Liver	3/12 (25%)	4/12 (33.3%)		
	Other	1/12 (8.3%)	2/12 (16.7%)	2/12 (16.7%)	
CTC number	High	6/19 (31.6%)	5/19 (26.3%)	4/19 (21.1%)	
	Low	2/19 (10.1%)	2/19 (10.1%)		
CTC cluster	Yes	6/19 (31.6%)	5/19 (26.3%)	4/19 (21.1%)	
	No	2/19 (10.1%)	2/19 (10.1%)		

* *p < 0.01*.

### Correlation Between CTC Gene Expression and OS or PFS in PDAC Patients

The correlation between the expression level of the 19 analyzed genes in CTCs and OS, OS1, and PFS was evaluated (Supplementary Tables 2, 3).

**Figure 6:**
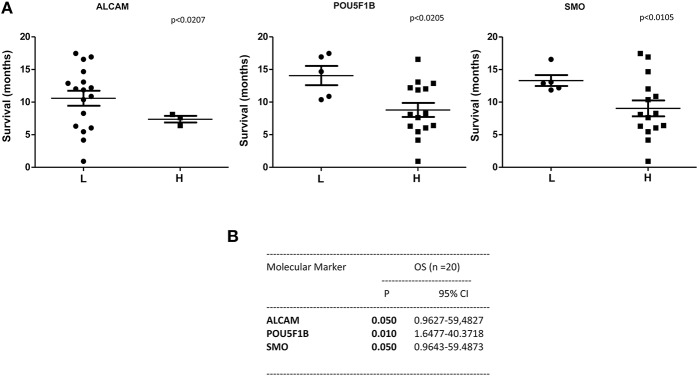
OS according to gene expression levels in CTCs from PDAC patients before chemotherapy. **(A)** Patients were stratified according to ALCAM, POU5F1B, and SMO gene expression levels in H (High) and L (Low) expressing. **(B)** OS evaluated according to the ALCAM, POU5F1B, and SMO gene expression levels by Univariate analysis. *p* < 0.05 was considered as statistically significant.

**Figure 7:**
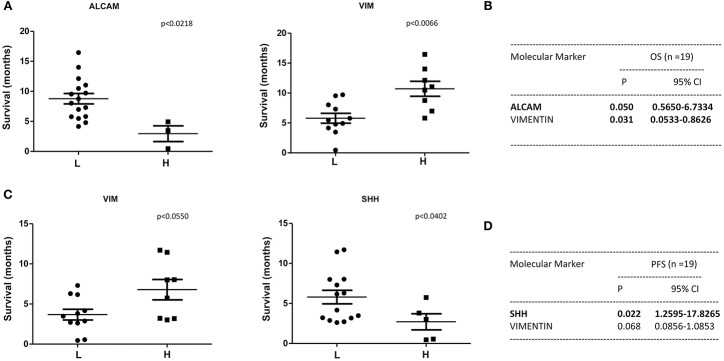
OS1 and PFS according to gene expression levels in CTCs from PDAC patients after chemotherapy. **(A)** OS1 (from start of 1st line) was evaluated in patients stratified into H (High) and L (Low) expressing, according to the ALCAM and VIM gene expression levels. **(B)** OS1 assessed according to the ALCAM and VIM gene expression levels by Univariate analysis. *p* < 0.05 was considered as statistically significant. **(C)** PFS (from start of 1st line) was evaluated in patients stratified into H (High) and L (Low) expressing, according to VIM and SHH gene expression levels. **(D)** PFS assessed according to the VIM and SHH gene expression levels by Univariate analysis.

A statistically significant correlation was found for the expression of ALCAM, POU5F1B, and SMO and OS in CTCs from PDAC patients (Figure 6A); similar results were obtained by Univariate analysis (Figure 6B). No positive correlation was found for the other analyzed genes (Supplementary Tables 2, 3). Similarly, after palliative chemotherapy, low ALCAM and high VIM levels were correlated with a longer OS1 (Figure 7A). These data were confirmed by Univariate analysis (Figure 7B). Regarding the PFS, high VIM, and low SHH levels were associated with a shorter PFS (Figure 7C). Similar results were obtained for SHH (*p* = 0.022) by Univariate Cox regression analysis (Figure 7D).

## Discussion

The majority of cancer patients dies from metastasis, regardless of chemotherapy approaches. Current cancer treatments are usually determined on primary tumor instead that on metastasis or cancer cells in blood circulation. Since distant metastases are considered to be the end-result of CTCs, the molecular characterization of these cells, including both cancer cells and tumacrophages (23), represents a promising approach to better evaluate the prognosis and select the therapy (30).

The identification of specific gene profiles and phenotypic changes occurring in CTCs could result in a better understanding of the metastatic process and lead to more effective and targeted therapeutic strategies. To our knowledge, very few reports have evaluated gene expression in blood CTCs. Moreover, this is the first study in which size-based CTC isolation method was coupled with ddPCR not only to confirm the CTCs phenotypes, but also to evaluate gene profiles.

Herein, we demonstrated the presence of single CTCs, atypical CTCs, and CTC clusters in the blood of PDAC patients before chemotherapy and 3 months later palliative first line chemotherapy. Our data are in agreement with recent data showing the presence of atypical CTCs, characterized by the expression of EPCAM, pan-CK, and CD45, in several cancer types. According to recent findings, they are hybrid cells deriving from the fusion of macrophages and tumor cells and for this reason, they are called tumacrophages. These cells, characterized by the expression of epithelial and hematopoietic markers as cytokeratins, CD45, and CD16, display progressive malignant behavior and tumorigenic abilities, contributing to the metastatic spread (23, 24).

High number of single CTCs and CTC clusters was captured in the blood of PDAC patients before and after chemotherapy. However, with regard to variations in the CTC number and cluster between before and after chemotherapy, in the majority of the patients, chemotherapy is not able to induce changes. Overall, we evidenced that increased CTC number represents a negative prognostic factor for poor OS and PFS in PDAC patients.

EMT process arises during pancreatic cancer progression generating highly tumorigenic stem cells, characterized by motility and invasiveness. Multiple studies reported that EMT/MET (mesenchymal epithelial transition) processes are common in CTCs (31). Due to the high heterogeneity and plasticity of metastatic PDAC cells, pancreatic cells often cross between an EMT to MET phenotype during the metastatic adaption (32). In this regard, Zhao et al. using the Can Patrol system, identified the presence of three distinct CTC phenotypes in PDAC patients: E-CTC, M-CTC, and E/M or H-CTCs. The CTC status correlated with lymph node invasion, TNM, and distant metastasis. In this study, KM survival analysis showed that patients with higher CTC count had significantly reduced OS and PFS compared with those showing lower CTC number (33). Moreover, by exploring the EMT phenomenon, it has been demonstrated that M-CTCs are associated with tumor progression and chemo-resistant cancer types (34). In this regard, we showed that E-CTCs and H-CSCs are found in PDAC patients before chemotherapy, whereas the M-CTCs subtype appears in chemotherapy-conditioned patients.

The identification of biomarkers in patients at high risk of poor prognosis might deserve additional/alternative therapeutic interventions also in advanced metastatic PDAC patients. In this regard, isolation of CTCs from blood sample holds the promise of diagnosing and molecular profiling cancers. CTCs are thought to represent the intravasation tumor stage between the primary cancer and its distant metastases. Thus, by ddPCR assay, the molecular profile of genes, involved in EMT, angiogenesis, stemness and pluripotency, Hedgehog, Notch, and Stat pathways, have been evaluated in CTCs from PDAC patients before and after palliative chemotherapy, and compared with normal pancreatic RNAs and PDAC biopsies. We found a strong up-regulation of ALCAM, IHH, and SHH, NOTCH1, EPCAM, PTCH1, PTCH2, POU5F1B, SMO and ZEB2, STAT3, and VIM genes in the CTCs compared to normal pancreatic samples and, in particular, respect to the biopsies. Moreover, using hierarchical clustering to analyze gene expression levels, two different gene clusters were identified in CTCs from PDAC patients, before and after palliative chemotherapy. In particular, the first group consisted of VEGFA, NOTCH1 and 2, EPCAM, IHH, CD44, ALCAM, VIM, SPARC, and POU5F1B genes whereas the other consisted of VEGFB, DHH and SHH, PTCH1 and 2, ZEB1 and 2, SMO, and STAT3 genes. After chemotherapy, the VEGFA, NOTCH1 and EPCAM phenotype was maintained, whereas a phenotype enriched of stemness and pluripotency genes such as VEGFB, CD44, ALCAM, NOTCH2, POU5F1B, PTCH1 and 2, STAT3, DHH, IHH and SMO, SPARC, and VIM, emerged. To better characterize the gene clustering based on positive correlation, multiple correlation index analysis was performed. A significant high correlation coefficient for several gene pairs was found allowing the identification of specific gene signatures in CTCs from PDAC patients: VEGFA/NOTCH1/EPCAM/IHH gene cluster before chemotherapy and VEGFB/POU5F1B/PTCH1/PTCH2/ALCAM/CD44 after palliative chemotherapy.

There is growing evidence that CTC population is extremely heterogeneous, with a small percentage of tumor cells, called cancer stem cells (CSCs) expressing CD44, ALCAM, POU5F1B (35), able to proliferate and form new tumors. The presence of CSCs in tumors is associated with aggressive disease and poor prognosis (35). We showed high ALCAM, POU5F1B, and SMO mRNA expression levels in CTCs that are predictive of poor OS in both untreated and treated PDAC patients. In addition, high SHH mRNA expression level in CTCs has been demonstrated to represent a negative factor for PFS.

CSCs express several markers such as CD44, CD166, POU5F1B, EPCAM, NOTCH1 (36), and STAT3 (37). In this regard, we observed during PDAC progression, after palliative chemotherapy, an enrichment in the CTCs of the expression levels of stemness and pluripotency genes, as CD44, ALCAM, EPCAM, NOTCH1, POU5F1B, PTCH1, or CSC drivers as VEGFB and STAT3.

The role of VEGFB in tumor progression remains controversial. In fact, although VEGFB overexpression predicts for increased distant metastasis and shorter OS in advanced cancers (38), it was also shown to delay tumor growth in a mouse model of pancreatic neuroendocrine tumorigenesis (39). VEGFB is a part of VEGFB/GSK-β3/PI3K-Akt/CD44 signaling pathway controlling stem cell renewal, differentiation and development (40). The CD44 induces the EMT and triggers the expression of POU5F1B/OCT-4 stem cell marker, through the AKT/GSK-3β/β-catenin pathway (41). POU5F1B/OCT-4 maintains pluripotency and self-renewal by interacting with STAT3 and the Hedgehog pathway. Its overexpression in PDAC induces cell proliferation, migration, invasion and gemcitabine resistance (GR) (42) and its expression correlates with the N1/M1 status and worse prognosis. In several KRAS mutated cancers, the CD44 molecule associates with ALCAM/CD166 promoting an aggressive phenotype that predicts worse outcome and increased risk of liver and lung metastasis (e.g., colon cancer) (29). In addition, high ALCAM levels are associated with poor survival, early tumor relapse (43) and chemoresistance (44). Regarding the Notch pathway, down-regulation of NOTCH1 reduces PDAC invasiveness (45), whereas NOTCH2 silencing reduces the expression of ZEB1, reverts the EMT phenotype, down-regulates the CSC marker expression (e.g., CD44) and decreases the invasiveness of GR-cells (46). In line with these findings, we showed the presence of CTCs expressing CD44/CD166 in PDAC patients with liver and lung metastasis. Thus, the analysis of gene expression levels in CTCs could permit to identify in KRAS mutated PDAC, patients expressing the CD44/CD166 phenotype associated with higher risk to develop lymph node invasion and distant metastasis. In addition, CTCs' characterization represents a non-invasive tool to identify, before palliative chemotherapy, patients predisposed to show GR-resistance, thus facilitating the design of individualized therapies.

Further support to our data, comes from *in vivo* experiments with metformin (met) treatment targeting several genes studied in our molecular analysis. In fact, met has been demonstrated to inhibit pancreatic intraepithelial neoplasia growth and the progression to PDAC, by reducing the CD44 or EPCAM stem cell marker expression in a transgenic mouse model (47).

On the contrary, we also showed that VIM expression positively correlates with a higher OS and PFS in PDAC patients after palliative first line chemotherapy. VIM, traditionally considered a marker of EMT, is also involved in angiogenesis, migration, invasion, metastasis, and drug-resistance (48, 49). For long time, high expression of VIM was only associated with poor prognosis in patients with different cancers (50). However, recently, high VIM expression was also correlated with a prolonged survival in endometrioid cancer patients (51) and better prognosis in ovarian cancer patients (46). This protective effect was explained by the role of VIM in the regulation of cancer cell-platinum resistance (52). Similarly to this study, it has been demonstrated that in Capan-1-GR cells the drug resistance is associated with VIM down-regulation (53). Thus, the increase of OS in patients showing CTCs with high VIM expression may be related to gemcitabine susceptibility in PDAC patients mainly treated with gemcitabine, alone or in combination with paclitaxel.

Overall, our data support the potential clinical value of CTCs from PDAC patients. CTC number, EMT/MET phenotype and molecular gene profile may contribute to the PDAC prognosis and therapy. Therefore, this study confirms that in PDAC high CTC number represents a negative prognostic factor, but also demonstrates that the identification of specific biomarkers could be useful to improve the prognosis and therapy in advanced PDAC patients. However, our pilot study includes a small sample size and large well-designed clinical trial is required to elucidate the real potential value of CTC gene profile in PDACs.

## Data Availability

Data generated or analyzed during this study, with the only exception of correlation matrix analysis for all studied genes, are included in this published article and its additional information files. The whole correlation matrix is available from the corresponding author.

## Ethics Statement

All patients gave their consent prior to blood draws and the local Ethical Committee of the Università Politecnica delle Marche–Azienda Ospedaliero-Universitaria Ospedali Riuniti Umberto I, Lancisi, Salesi, Ancona, Italy, approved the study. The patients/participants provided their written informed consent to participate in this study.

## Author Contributions

CA contributed to the acquisition, analysis, interpretation of the data, and drafted the manuscript. MM and MN performed acquisition and interpretation of the data. FM and FB revised the manuscript. FP and OM were responsible for analysis. AB and RG participated to the acquisition of the data. RB contributed to the conception of the work and revised the manuscript. GS designed the work and drafted the manuscript.

### Conflict of Interest Statement

The authors declare that the research was conducted in the absence of any commercial or financial relationships that could be construed as a potential conflict of interest.

## References

[1] Siegel RL Miller KD Jemal A Cancer statistics, 2019. CA Cancer J Clin. (2019) 69:7–34. 10.3322/caac.21551 30620402

[2] Rawla P Sunkara T Gaduputi V. Epidemiology of pancreatic cancer: global trends, etiology and risk factors. World J Oncol. (2019) 10:10–27. 10.14740/wjon1166 30834048 PMC6396775

[3] Hidalgo M. Pancreatic cancer. N Engl J Med. (2010) 362:1605–17. 10.1056/NEJMra0901557 20427809

[4] Von Hoff DD Ramanathan RK Borad MJ Laheru DA Smith LS Wood TE. Gemcitabine plus nab-paclitaxel is an active regimen in patients with advanced pancreatic cancer: a phase I/II trial. J Clin Oncol. (2011) 29:4548–54. 10.1200/JCO.2011.36.5742 21969517 PMC3565012

[5] Conroy T Desseigne F Ychou M Bouché O Guimbaud R Bécouarn Y. FOLFIRINOX versus gemcitabine for metastatic pancreatic cancer. N Engl J Med. (2011) 364:1817–25. 10.1056/NEJMoa1011923 21561347

[6] Jeong KY Kim EK Park MH Kim HM. Perspective on cancer therapeutics utilizing analysis of circulating tumor cells. Diagnostics. (2018) 8:23. 10.3390/diagnostics8020023 29641512 PMC6023425

[7] Fabisiewicz A Grzybowska E. CTC clusters in cancer progression and metastasis. Med Oncol. (2017) 34:12. 10.1007/s12032-016-0875-0 28012133

[8] Khoja L Backen A Sloane R Menasce L Ryder D Krebs M. A pilot study to explore circulating tumour cells in pancreatic cancer as a novel biomarker. Br J Cancer. (2012) 106:508–16. 10.1038/bjc.2011.545 22187035 PMC3273340

[9] Nicolazzo C Colangelo L Corsi A Carpino G Gradilone A Sonato C. Liquid biopsy in rare cancers: lessons from hemangiopericytoma. Anal Cell Pathol. (2018) 2018:9718585. 10.1155/2018/9718585 29707475 PMC5863319

[10] Chudasama D Barr J Beeson J Beddow E McGonigle N Rice A. Detection of circulating tumour cells and survival of patients with non-small cell lung cancer. Anticancer Res. (2017) 37:169–73. 10.21873/anticanres.11302 28011487

[11] Awe JA Saranchuk J Drachenberg D Mai S. Filtration-based enrichment of circulating tumor cells from all prostate cancer risk groups. Urol Oncol. (2017) 35:300–9. 10.1016/j.urolonc.2016.12.008 28202223

[12] Fina E Necchi A Bottelli S Reduzzi C Pizzamiglio S Iacona C. Detection of circulating tumour cells in urothelial cancers and clinical correlations: comparison of two methods. Dis Markers. (2017) 2017:3414910. 10.1155/2017/3414910 28321147 PMC5340956

[13] Kulasinghe A Perry C Jovanovic L Nelson C Punyadeera C. Circulating tumour cells in metastatic head and neck cancers. Int J Cancer. (2015) 136:2515–23. 10.1002/ijc.29108 25111594

[14] Kulemann B Pitman MB Liss AS Valsangkar N Fernández-Del Castillo C Lillemoe KD. Circulating tumor cells found in patients with localized and advanced pancreatic cancer. Pancreas. (2015) 44:547–50. 10.1097/MPA.0000000000000324 25822154

[15] Iwanicki-Caron I Basile P Toure E Antonietti M Lecleire S Di Fiore A. Usefulness of circulating tumor cell detection in pancreatic adenocarcinoma diagnosis. Am J Gastroenterol. (2013) 108:152–5. 10.1038/ajg.2012.367 23287955

[16] Ankeny JS Court CM Hou S Li Q Song M Wu D. Circulating tumour cells as a biomarker for diagnosis and staging in pancreatic cancer. Br J Cancer. (2016) 114:1367–75. 10.1038/bjc.2016.121 27300108 PMC4984454

[17] Nagrath S Sequist LV Maheswaran S Bell DW Irimia D Ulkus L. Isolation of rare circulating tumour cells in cancer patients by microchip technology. Nature. (2007) 450:1235–9. 10.1038/nature06385 18097410 PMC3090667

[18] Poruk KE Valero V III Saunders T Blackford AL Griffin JF Poling J. Circulating tumor cell phenotype predicts recurrence and survival in pancreatic adenocarcinoma. Ann Surg. (2016) 264:1073–81. 10.1097/SLA.0000000000001600 26756760 PMC4936958

[19] Han L Chen W Zhao Q. Prognostic value of circulating tumor cells in patients with pancreatic cancer: a meta-analysis. Tumour Biol. (2014) 35:2473–80. 10.1007/s13277-013-1327-5 24218336

[20] Okubo K Uenosono Y Arigami T Mataki Y Matsushita D Yanagita S Corrigendum to “Clinical impact of circulating tumor cells and therapy response in pancreatic cancer” [43 (6) (2017) 1050-1055]. Eur J Surg Oncol. (2018) 44:860 10.1016/j.ejso.2018.03.015 29605162

[21] Chang MC Chang YT Chen JY Jeng YM Yang CY Tien YW. Clinical significance of circulating tumor microemboli as a prognostic marker in patients with pancreatic ductal adenocarcinoma. Clin Chem. (2016) 62:505–13. 10.1373/clinchem.2015.248260 26861552

[22] Torphy RJ Tignanelli CJ Kamande JW Moffitt RA Herrera Loeza SG Soper SA. Circulating tumor cells as a biomarker of response to treatment in patient-derived xenograft mouse models of pancreatic adenocarcinoma. PLoS ONE. (2014) 9:e89474. 10.1371/journal.pone.0089474 24586805 PMC3929698

[23] Gast CE Silk AD Zarour L Riegler L Burkhart JG Gustafson KT. Cell fusion potentiates tumor heterogeneity and reveals circulating hybrid cells that correlate with stage and survival. Sci Adv. (2018) 4:eaat7828. 10.1126/sciadv.aat7828 30214939 PMC6135550

[24] Zhang Y Zhou N Yu X Zhang X Li S Lei Z. Tumacrophage: macrophages transformed into tumor stem-like cells by virulent genetic material from tumor cells. Oncotarget. (2017) 8:82326–43. 10.18632/oncotarget.19320 29137267 PMC5669893

[25] Hidalgo M Cascinu S Kleef J Labianca R Lohr JM Neoptolemos J Addressing the challenges of pancreatic cancer: Future directions fro improving outcomes. Pancreatology. (2015) 15:8–18. 10.1016/j.pan.2014.10.001 25547205

[26] Oliveira-Cuhna M Newman WG Siriwardena AK Epidermal growth factor receptor in pancreatic cancer. Cancers. (2011) 3:1513–6. 10.3390/cancers3021513 24212772 PMC3757375

[27] Khan MA Azim S Zubair H Bhardwaj A Patel GK Khushman M. Molecular drivers of pancreatic cancer pathogenesis: looking inward to move forward. Int J Mol Sci. (2017) 18:E779. 10.3390/ijms18040779 28383487 PMC5412363

[28] Aras S Zaidi MR. TAMless traitors: macrophages in cancer progression and metastasis. Br J Cancer. (2017) 117:1583–91. 10.1038/bjc.2017.356 29065107 PMC5729447

[29] Ribeiro KB da Silva Zanetti J Ribeiro-Silva A Rapatoni L de Oliveira HF da Cunha Tirapelli DP KRAS mutation associated with CD44/CD166 immuno expression as predictors of worse outcome in metastatic colon cancer. Cancer Biomark. (2016) 16:513–21. 10.3233/CBM-160592 27062566 PMC13016524

[30] Sergeant G van Eijsden R Roskams T Van Duppen V Topal B. Pancreatic cancer circulating tumour cells express a cell motility gene signature that predicts survival after surgery. BMC Cancer. (2012) 12:527. 10.1186/1471-2407-12-527 23157946 PMC3599097

[31] Harouaka R Kang Z Zheng SY Cao L. Circulating tumor cells: advances in isolation and analysis, and challenges for clinical applications. Pharmacol Ther. (2014) 141:209–21. 10.1016/j.pharmthera.2013.10.004 24134902 PMC3947247

[32] Samain R Jean C Bousquet C Pancreatic cancer cell invasion: mesenchymal switch or just hitchhiking? Transl Cancer Res. (2016) 5:S1093–7. 10.21037/tcr.2016.11.09

[33] Zhao XH Wang ZR Chen CL Di L Bi ZF Li ZH. Molecular detection of epithelial-mesenchymal transition markers in circulating tumor cells from pancreatic cancer patients: potential role in clinical practice. World J Gastroenterol. (2019) 25:138–50. 10.3748/wjg.v25.i1.138 30643364 PMC6328963

[34] Satelli A Mitra A Brownlee Z Xia X Bellister S Overman MJ. Epithelial-mesenchymal transitioned circulating tumor cells capture for detecting tumor progression. Clin Cancer Res. (2015) 21:899–906. 10.1158/1078-0432.CCR-14-0894 25516888 PMC4334736

[35] Nguyen LV Vanner R Dirks P Eaves CJ. Cancer stem cells: an evolving concept. Nat Rev Cancer. (2012) 12:133–43. 10.1038/nrc3184 22237392

[36] Marhaba R Klingbeil P Nuebel T Nazarenko I Buechler MW Zoeller M. CD44 and EPCAM: cancer-initiating cell markers. Curr Mol Med. (2008) 8:784–804. 10.2174/156652408786733667 19075676

[37] Deschênes-Simard X Parisotto M Rowell MC Le Calvé B Igelmann S Moineau-Vallée K. Circumventing senescence is associated with stem cell properties and metformin sensitivity. Aging Cell. (2019) 18:e12889. 10.1111/acel.12889 30614183 PMC6413657

[38] Yang X Zhang Y Hosaka K Andersson P Wang J Tholander F. VEGF-B promotes cancer metastasis through a VEGF-A-independent mechanism and serves as a marker of poor prognosis for cancer patients. Proc Natl Acad Sci USA. (2015) 112:E2900–9. 10.1073/pnas.1503500112 25991856 PMC4460438

[39] Albrecht I Kopfstein L Strittmatter K Schomber T Falkevall A Hagberg CE. Suppressive effects of vascular endothelial growth factor-B on tumor growth in a mouse model of pancreatic neuroendocrine tumorigenesis. PLoS ONE. (2010) 5:e14109. 10.1371/journal.pone.0014109 21124841 PMC2991338

[40] Seino S Shigeishi H Hashikata M Higashikawa K Tobiume K Uetsuki R CD44(high) /ALDH1(high) head and neck squamous cell carcinoma cells exhibit mesenchymal characteristics and GSK3 β-dependent cancer stem cell properties. J Oral Pathol Med. (2016) 45:180–8. 10.1111/jop.12348 26399460

[41] Park NR Cha JH Jang JW Bae SH Jang B Kim JH. Synergistic effects of CD44 and TGF-β1 through AKT/GSK-3β/β-catenin signaling during epithelial-mesenchymal transition in liver cancer cells. Biochem Biophys Res Commun. (2016) 477:568–74. 10.1016/j.bbrc.2016.06.077 27320862

[42] Wang D Zhu H Zhu Y Liu Y Shen H Yin R Retraction notice to “CD133+/CD44+/Oct4+/Nestin+ stem-like cells isolated from Panc-1 cell line may contribute to multi-resistance and metastasis of pancreatic cancer”. Acta Histochem. (2018) 120:302 10.1016/j.acthis.2018.03.005 29598902

[43] Kahlert C Weber H Mogler C Bergmann F Schirmacher P Kenngott HG. Increased expression of ALCAM/CD166 in pancreatic cancer is an independent prognostic marker for poor survival and early tumour relapse. Br J Cancer. (2009) 101:457–64. 10.1038/sj.bjc.6605136 19603023 PMC2720248

[44] Hong X Michalski CW Kong B Zhang W Raggi MC Sauliunaite D. ALCAM is associated with chemoresistance and tumor cell adhesion in pancreatic cancer. J Surg Oncol. (2010) 101:564–9. 10.1002/jso.21538 20461761

[45] Wang Z Banerjee S Li Y Rahman KM Zhang Y Sarkar FH. Down-regulation of notch-1 inhibits invasion by inactivation of nuclear factor-kappaB, vascular endothelial growth factor, and matrix metalloproteinase-9 in pancreatic cancer cells. Cancer Res. (2006) 66:2778–84. 10.1158/0008-5472.CAN-05-4281 16510599

[46] Shah AN Summy JM Zhang J Park SI Parikh NU Gallick GE. Development and characterization of gemcitabine-resistant pancreatic tumor cells. Ann Surg Oncol. (2007) 14:3629–37. 10.1245/s10434-007-9583-5 17909916

[47] Mohammed A Janakiram NB Brewer M Ritchie RL Marya A Lightfoot S. Antidiabetic drug metformin prevents progression of pancreatic cancer by targeting in part cancer stem cells and mTOR signaling. Transl Oncol. (2013) 6:649–59. 10.1593/tlo.13556 24466367 PMC3890699

[48] Satelli A Li S. Vimentin in cancer and its potential as a molecular target for cancer therapy. Cell Mol Life Sci. (2011) 68:3033–46. 10.1007/s00018-011-0735-1 21637948 PMC3162105

[49] Huo Y Zheng Z Chen Y Wang Q Zhang Z Deng H. Downregulation of vimentin expression increased drug resistance in ovarian cancer cells. Oncotarget. (2016) 7:45876–88. 10.18632/oncotarget.9970 27322682 PMC5216767

[50] Liu LG Yan XB Xie RT Jin ZM Yang Y. Stromal expression of vimentin predicts the clinical outcome of stage II colorectal cancer for high-risk patients. Med Sci Monit. (2017) 23:2897–905. 10.12659/MSM.904486 28611349 PMC5479442

[51] Zou S Sun H Fan L Xiao X Gong L Zhu J Prognostic indicators in patients with early stage endometrioid adenocarcinoma: a retrospective case-control study of 523 patients. Int J Clin Exp Med. (2017) 10:3692–8.

[52] Szubert S Koper K Dutsch-Wicherek MM Jozwicki W. High tumor cell vimentin expression indicates prolonged survival in patients with ovarian malignant tumors. Ginekol Pol. (2019) 90:11–9. 10.5603/GP.2019.0003 30756366

[53] Avan A Quint K Nicolini F Funel N Frampton AE Maftouh M. Enhancement of the antiproliferative activity of gemcitabine by modulation of c-Met pathway in pancreatic cancer. Curr Pharm Des. (2013) 19:940–50. 10.2174/138161213804547312 22973962

