# Supplementary material for: Expression Profiling of Circulating Tumor Cells in Pancreatic Ductal Adenocarcinoma Patients: Biomarkers Predicting Overall Survival
Source: Front Oncol. 2019 Sep 10;9:874. doi: 10.3389/fonc.2019.00874 (PMC6746928; doi:10.3389/fonc.2019.00874)
Supplement: Supplementary file 1 [file Table_1.DOCX]

**Supplementary Table 1 Kaplan-Meier analysis for OS, OS1 and PFS according to age, sex and TNM**

-------------------------------------------------------------------------------------

Parameter OS (n = 20) OS1 (n = 19) PFS (n = 19)

----------------------------------------------------------------

P P P

-------------------------------------------------------------------------------------

Age 0.3655 0.2903 0.2267

Sex 0.7948 0.4011 **0.0470***

T 0.3076 0.3702 0.5763

N 0.6074 0.6638 0.3287

M 0.7254 0.5479 0.9165

--------------------------------------------------------------------------------------

*p<0.05

OS: overall survival (time between the diagnosis and the death or lost-at-follow-up visit); OS1: overall survival 1 (the time between the 1^st^ cycle of chemotherapy and death or lost-at-follow-up visit); PFS (the time between the 1^st^ cycle of chemotherapy and the first radiological progression or lost-at-follow-up-visit).
