# Supplementary material for: Expression Profiling of Circulating Tumor Cells in Pancreatic Ductal Adenocarcinoma Patients: Biomarkers Predicting Overall Survival
Source: Front Oncol. 2019 Sep 10;9:874. doi: 10.3389/fonc.2019.00874 (PMC6746928; doi:10.3389/fonc.2019.00874)
Supplement: Supplementary file 2 [file Table_2.DOCX]

**Supplementary Table 2 Kaplan-Meier analysis for OS or OS1 and the expression levels of the analyzed molecular markers before and after palliative chemotherapy.**

---------------------------------------------------------------------------------------------------------------------------

Molecular Marker OS (n = 20) OS1 (n = 19)

-------------------------------------------------- --------------------------------------------

P HR 95% CI P HR 95% CI

----------------------------------------------------------------------------------------------------------------------------

CD44 0.5585 0.4187

**ALCAM 0.0500* 0.4272 0.0071-1.2570**  **0.0297* 0.2262 0.0023-0.7299**

VEGFA 0.5623 0.5246

VEGFB 0.1489 0.3422

ZEB1 0.6505 0.4989

ZEB2 0.1325 0.3039

**POU5F1B** **0.0034* 0.2236 0.0355-0.5158** 0.0699

**VIM** 0.9571 **0.0286* 3.2761 1.1791-19.6491**

DHH 0.2168 0.2070

IHH 0.9482 0.5159

SHH 0.4217 0.5117

PITCH1 0.0987 0.0665

PITCH2 0.3670 0.4200

**SMO** **0.0253* 0.1469 0.0731-0.8418** 0.0848

NOTCH1 0.5628 0.4480

NOTCH2 0.8671 0.2090

SPARC 0.0963 0.1690

STAT3 0.2019 0.2353

EPCAM 0.2968 0.3580

CA19.9 0.3029 0.6173

CEA 0.3263 0.3738

------------------------------------------------------------------------------------------------------------------------

*p<0.05
