# Supplementary material for: Expression Profiling of Circulating Tumor Cells in Pancreatic Ductal Adenocarcinoma Patients: Biomarkers Predicting Overall Survival
Source: Front Oncol. 2019 Sep 10;9:874. doi: 10.3389/fonc.2019.00874 (PMC6746928; doi:10.3389/fonc.2019.00874)
Supplement: Supplementary file 3 [file Table_3.docx]

**Supplementary Table 3 Kaplan-Meier analysis for PFS and molecular marker expression levels after chemotherapy.**

------------------------------------------------------------------------------

Molecular Marker PFS (n = 19)

-----------------------------------------------

P HR 95% CI

-------------------------------------------------------------------------------

CD44 0.8531

ALCAM 0.3559

VEGFA 0.2221

VEGFB 0.3755

ZEB1 0.8744

ZEB2 0.9824

POU5F1B 0.2274

PTPRC 0.3170

VIM **0.0500* 2.9489 0.9789-14.3145**

DHH 0.9282

IHH 0.6940

SHH **0.0121* 0.2563 0.0257-0.6370**

PITCH1 0.2217

PITCH2 0.9303

SMO 0.0807

NOTCH1 0.2221

NOTCH2 0.1206

SPARC 0.2077

STAT3 0.9428

EPCAM 0.6726

----------------------------------------------------------------------------------

*p<0.05
